# P-1346. Comparative Effectiveness of Parenteral Daptomycin Versus Vancomycin Among Patients with Methicillin-resistant Staphylococcus aureus (MRSA) Endogenous endophthalmitis: A Retrospective Analysis

**DOI:** 10.1093/ofid/ofaf695.1534

**Published:** 2026-01-11

**Authors:** Silvana Ribeiro Papp, Zinaida Perciuleac, Paddy Ssentongo, Siddartha Guru, Alex Jakubiak, Sulochana Khadka, Poonam Bai, Anas Atrash

**Affiliations:** UPMC, Dover, PA; Penn State Health Milton S. Hershey Medical Center, Hershey, Pennsylvania; Penn State Health Milton S. Hershey Medical Center, Hershey, Pennsylvania; Penn State Health Milton S. Hershey Medical Center, Hershey, Pennsylvania; Drexel University, Harrisburg, Pennsylvania; UPMC, Dover, PA; Penn State Health Milton S. Hershey Medical Center, Hershey, Pennsylvania; UPMC, Dover, PA

## Abstract

**Background:**

Endogenous endophthalmitis is a rare complication of Staphylococcus aureus, including its methicillin-resistant (MRSA), presents a significant clinical challenge due to its potential for irreversible vision. Vancomycin and daptomycin are commonly used systemic and intravitreal antibiotics; however, their comparative effectiveness in treating MRSA endogenous endophthalmitis remains unclear. This study aims to compare the efficacy and safety of daptomycin versus vancomycin in MRSA bacteremia with endophthalmitis.

**Figure 1:**
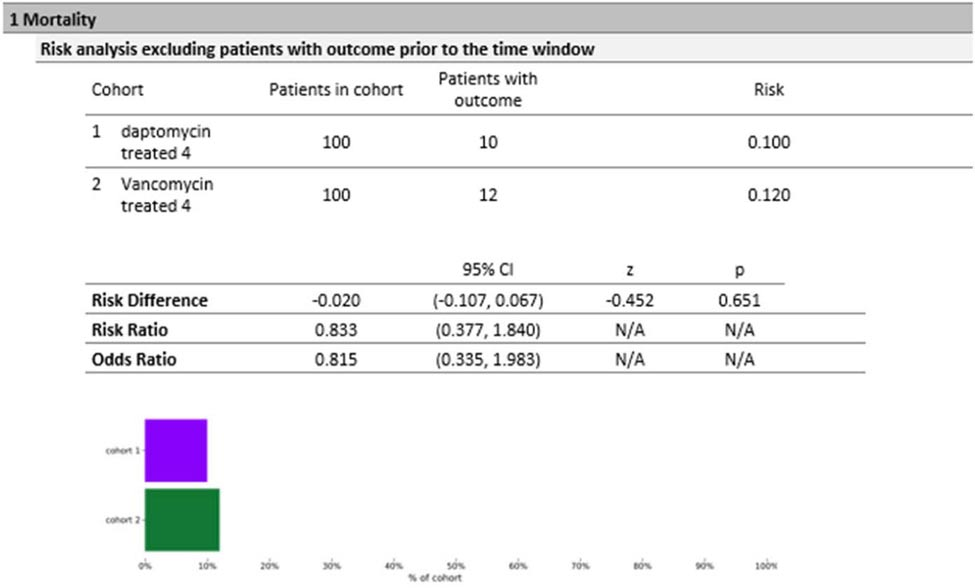
Mortality In this matched cohort analysis of 200 patients with MRSA endophthalmitis (100 treated with daptomycin, 100 with vancomycin), mortality rates were similar—10% for daptomycin vs. 12% for vancomycin (risk difference -0.020; 95% CI: -0.107, 0.067; p = 0.651).

**Figure 2:**
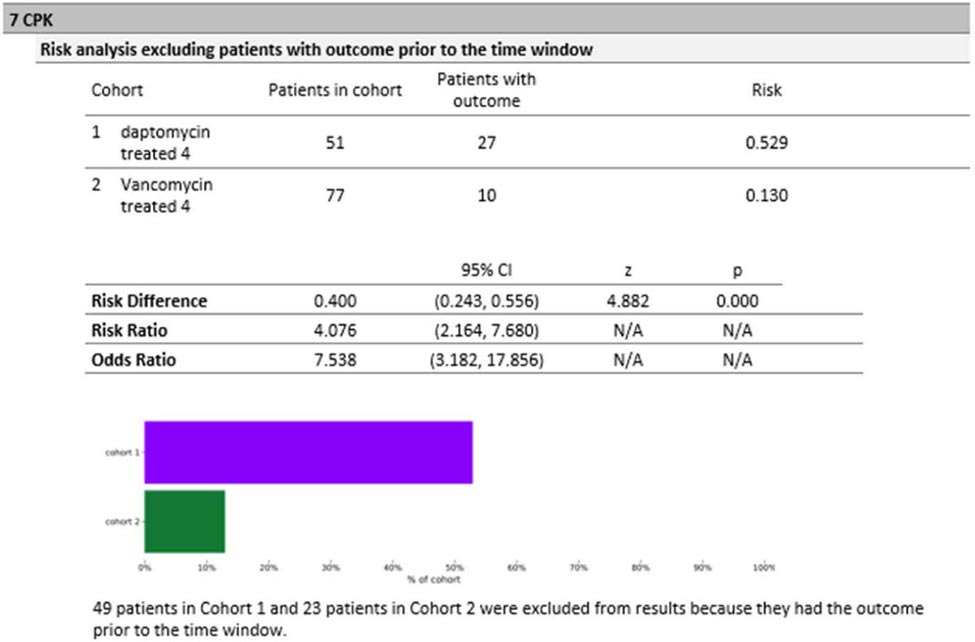
CPK levels CPK elevation, indicating muscle toxicity, was much higher with daptomycin (52.9% vs. 13.0%; risk difference 0.400; 95% CI: 0.243, 0.556; p < 0.0001)

**Methods:**

A retrospective observational cohort study was conducted using the TriNetX Research Collaborative Network. Adults diagnosed with MRSA endophthalmitis were included into daptomycin and vancomycin treatment groups. Propensity score matching was performed to balance covariates between groups. Statistical analyses included Kaplan-Meier survival curves, Cox proportional hazard regression, and log-rank tests.

**Results:**

In this matched cohort analysis of 200 patients with MRSA endophthalmitis (100 treated with daptomycin, 100 with vancomycin), mortality rates were similar—10% for daptomycin vs. 12% for vancomycin (risk difference -0.020; 95% CI: -0.107, 0.067; p = 0.651), as were rates of blindness (13.9% vs. 13.5%; risk difference 0.004; 95% CI: -0.108, 0.115; p = 0.947), endocarditis (11.6% vs. 11.4%; risk difference 0.003; 95% CI: -0.092, 0.097; p = 0.956). Septic shock (27.0% vs. 18.5%) and bacteremia were more frequent with daptomycin, but not significantly. CPK elevation was higher in daptomycin (52.9% vs. 13.0%; risk difference 0.400; 95% CI: 0.243, 0.556; p < 0.0001), and CKD stage 4/5 occurred in 12.2% of daptomycin patients versus 0% for vancomycin (risk difference 0.122; 95% CI: 0.051, 0.193; p = 0.001). Notably, embolic stroke occurred in 0% of daptomycin patients compared to 10.4% vancomycin (risk difference -0.104; 95% CI: -0.165, -0.043; p = 0.001). Baseline characteristics were well-matched, including mean age (53.5 vs. 54 years), diabetes (55% vs. 45%), and mean BMI (29.2 vs. 28.3).

**Conclusion:**

Daptomycin and vancomycin provide comparable effectiveness for major clinical outcomes in MRSA endophthalmitis, but their safety profiles differ.

**Disclosures:**

All Authors: No reported disclosures

